# Klebsiella Pyogenic Liver Abscess as the Initial Presentation of Undiagnosed Type 2 Diabetes Mellitus: A Case Report and Review

**DOI:** 10.7759/cureus.110957

**Published:** 2026-06-16

**Authors:** Jewairia Alajab, Namrata Vedagarbha, Mia K Sooch, Moayyed Hussein, Abdelwahab Ahmed, Mustafa Marzoug

**Affiliations:** 1 Internal Medicine, Detroit Medical Center, Wayne State University School of Medicine, Detroit, USA; 2 Internal Medicine, Wayne State University School of Medicine, Detroit, USA

**Keywords:** bacteremia, diabetes mellitus type 2, hyperglycemia, klebsiella pneumoniae, pyogenic liver abscess (pla)

## Abstract

Type 2 diabetes mellitus is associated with impaired innate immunity and increased susceptibility to invasive infections, which may occasionally represent the first clinical manifestation of underlying metabolic disease. Pyogenic liver abscess (PLA), particularly due to *Klebsiella pneumoniae*, has a well-established association with diabetes and may unmask previously undiagnosed hyperglycemia. We report the case of a 40-year-old male patient with no known past medical history who presented with four days of fever, lethargy, and vomiting. On admission, he was febrile to 39.4°C and tachycardic, with laboratory evidence of leukocytosis. Initial evaluation revealed marked hyperglycemia with glucose levels in the 400s and an HbA1c of 13.1%, consistent with previously undiagnosed type 2 diabetes mellitus. Computed tomography (CT) of the abdomen and pelvis demonstrated a necrotic mass in the right hepatic lobe, along with a smaller adjacent lesion, concerning a PLA. Blood cultures grew *Klebsiella pneumoniae* in multiple sets, confirming bacteremia. The patient underwent CT-guided aspiration and drainage of the hepatic abscess with placement of a pigtail catheter, yielding purulent material that also grew *Klebsiella pneumoniae*. His hospital course was complicated by sepsis and acute hypoxic respiratory failure requiring intensive care unit admission. He was treated with intravenous ceftriaxone and metronidazole, resulting in gradual clinical improvement and clearance of bacteremia. Concurrently, insulin therapy was initiated, and the patient received diabetes education for the management of newly diagnosed diabetes. This case highlights invasive infection as a potential initial presentation of undiagnosed diabetes mellitus. Hyperglycemia impairs neutrophil function and host defense, predisposing patients to severe infections such as *Klebsiella* liver abscesses. Recognition of this relationship is essential, as identification and management of underlying diabetes are critical to optimizing outcomes and preventing recurrence. This case underscores the importance of screening for diabetes in patients presenting with severe or atypical infections and illustrates how metabolic disease may first manifest through life-threatening infectious complications.

## Introduction

Pyogenic liver abscess (PLA) is a high-mortality infection of the hepatic parenchyma [1]. While historically linked to enteric organisms via biliary or portal venous spread, the last three decades have seen the emergence of hypervirulent *Klebsiella pneumoniae* (hvKp) as a newly appreciated causative pathogen [2]. *Klebsiella pneumoniae* liver abscess (KLA) was initially recognized as an endemic syndrome in East and Southeast Asia; however, its global incidence is increasing, with hvKp now identified as an important pathogen in Europe and the United States [2]. 

These hypervirulent strains, most commonly expressing K1 and K2 capsular serotypes, are thought to translocate from the gastrointestinal tract to the liver, potentially via the portal venous system. This allows them to evade clearance by Kupffer cells, the liver’s resident macrophages [2]. KLA may be further complicated by metastatic spread to distant sites, including the lungs, central nervous system, and eyes, a phenomenon termed invasive *Klebsiella pneumoniae* liver abscess syndrome (IKPLAS) [3]. 

Diabetes mellitus is the most significant risk factor for KLA. A large retrospective study found that nearly half of patients with KLA had concurrent diabetes mellitus, a significantly higher proportion than in non-*Klebsiella *liver abscesses [4]. A recent meta-analysis confirmed that diabetes confers a nearly twofold increased risk of IKPLAS and that poor glycemic control, rather than bacterial virulence factors alone, is a primary driver of invasive disease [3,5]. Given that an estimated half of individuals living with diabetes worldwide remain undiagnosed, invasive infections may represent the first clinical manifestation of underlying hyperglycemia [6]. 

The pathophysiologic basis for this susceptibility is multifactorial and centers on hyperglycemia-mediated immune dysfunction. Chronic hyperglycemia impairs multiple aspects of the innate immune response, including neutrophil chemotaxis, phagocytosis, and intracellular microbial killing [7,8]. In patients with poorly controlled type 2 diabetes, phagocytosis of K1/K2 serotype *Klebsiella pneumoniae* by neutrophils is significantly impaired compared to both well-controlled diabetic patients and healthy controls [9]. Furthermore, elevated glucose concentrations directly stimulate capsular polysaccharide biosynthesis in virulent *Klebsiella pneumoniae* strains, enhancing resistance to opsonophagocytic killing [10]. 

Hyperglycemia also promotes aberrant neutrophil extracellular trap formation through NADPH oxidase-mediated reactive oxygen species generation, paradoxically reducing the oxidative burst capacity required for effective pathogen clearance [11]. Together, these mechanisms create a “permissive environment” in which uncontrolled diabetes and bacterial virulence converge to facilitate life-threatening invasive disease. 

## Case presentation

A 40-year-old Arabic-speaking male patient with no known past medical history presented with four days of fever, lethargy, vomiting, and headache. He had immigrated from Mauritania two years prior and denied prior diagnoses of diabetes or other chronic illnesses. 

On arrival, he was febrile at 39.4°C and tachycardic with a heart rate in the 140s (beats per minute), while maintaining oxygen saturation above 95% on room air. Initial laboratory evaluation was notable for leukocytosis, thrombocytopenia, acute kidney injury, transaminitis, direct hyperbilirubinemia with elevated total bilirubin, and elevated direct bilirubin (Table 1). Serum glucose was markedly elevated in the 400s (reference range: 70-140 mg/dL), and HbA1c was 13.1%. These findings, along with the patient's age and race, are consistent with previously undiagnosed type 2 diabetes mellitus.

**Table 1:** Summary of abnormal laboratory values on admission

	Patient Value	Reference Range
WBC Count	13.4 x 10^9^/L	4.5-11 x 10^9^/L
Platelet count	78 x 10^9^/L	150-400 x10^9^/L
Blood urea nitrogen	31 mg/dL	10-20 mg/dL
Serum creatinine	1.39 mg/dL	0.6-1.2 mg/dL
Aspartate aminotransferase	96 U/L	10-40 U/L
Alanine transaminase	91 U/L	10-40 U/L
Total bilirubin	3.81 mg/dL	0.1-1.2 mg/dL
Direct bilirubin	2.19 mg/dL	0.0-0.3 mg/dL

Computed tomography (CT) of the abdomen and pelvis with contrast demonstrated a 5.0 × 2.5 cm necrotic mass in the dome of the right hepatic lobe, along with a smaller 2 cm solid lesion in the same region, raising concern for either infectious or neoplastic etiology (Figure 1). CT is highly sensitive for detecting visceral parenchymal abscesses, as it identifies low-density masses and extraluminal gas, both of which are suggestive of abscess formation. In this patient, air foci were noted on the medial aspect of the right hepatic dome. Blood cultures obtained on admission grew *Klebsiella pneumoniae* in all bottles. However, there was a limitation in additional microbiology details, as isolate testing for extended-spectrum beta-lactamase (ESBL) or carbapenemase production was not performed, nor were hypervirulent features such as K1/K2 serotype, string test, or virulence genes assessed.

**Figure 1:**
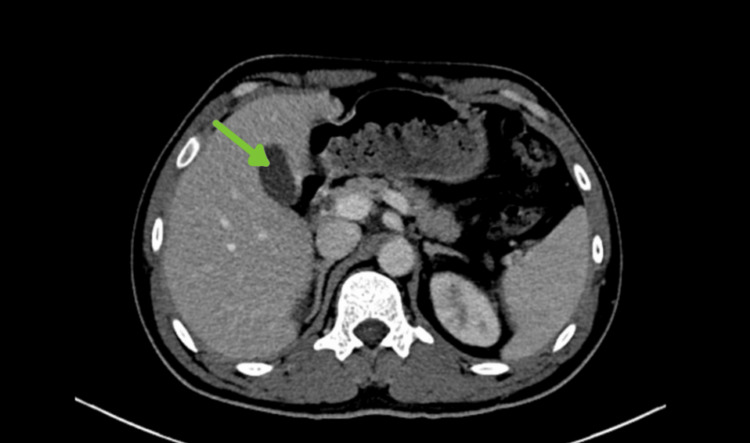
Contrast-enhanced abdominal CT demonstrating a hypodense right hepatic lobe lesion with central necrosis and peripheral rim enhancement, consistent with a pyogenic liver abscess.

On hospital day 2, the patient underwent CT-guided aspiration of the hepatic lesion by interventional radiology, yielding purulent material. Approximately 4-5 mL of fluid was aspirated; the low volume was due to multiloculation of the collection. An 8-French pigtail catheter was placed for ongoing drainage. Culture of the aspirated fluid also grew *Klebsiella pneumoniae*, confirming the diagnosis of PLA as the source of bacteremia. Cytology was sent, but no solid lesion was identified for biopsy. 

Immediately following the procedure, the patient developed acute hypoxic respiratory failure with tachypnea and increased oxygen requirements, necessitating transfer to the medical intensive care unit (MICU). This was attributed to pulmonary vascular congestion and reduced lung volume observed on chest X-ray. B-type natriuretic peptide (BNP) was slightly elevated at 125 pg/mL (reference range: <100 pg/mL). He required up to 8 L oxygen via a simple face mask but did not require mechanical ventilation. A transthoracic echocardiogram showed no evidence of endocarditis or cardiac dysfunction, and revealed normal left ventricular size and thickness with an ejection fraction of 70%. Ophthalmologic evaluation was unremarkable, with no evidence of endogenous endophthalmitis. Neurologic examination was nonfocal, and a CT of the head was unremarkable. 

The patient was initially treated with broad-spectrum antibiotics, which were subsequently narrowed to intravenous ceftriaxone (2 g daily) and metronidazole (500 mg every 12 hours) based on culture sensitivities and infectious disease recommendations. According to recent guidelines, ceftriaxone plus metronidazole is the first-line regimen (Grade 1A) for intra-abdominal infections, including liver abscess. Repeat blood cultures obtained on hospital days 2 and 4 were negative, indicating clearance of bacteremia. 

Follow-up imaging demonstrated a multiloculated abscess in the medial right hepatic dome measuring 6.7 × 4.1 × 3.8 cm, with a second smaller lesion measuring approximately 2.1 × 1.5 × 1.8 cm. Despite a slight interval increase in size, repeat drainage was deemed not feasible due to challenging access and proximity to critical vascular structures. Surgical consultation similarly determined that operative intervention was not indicated. 

Additional imaging findings included rectosigmoid wall thickening and narrowing, raising concern for a possible neoplastic process. However, subsequent colonoscopy revealed only small internal hemorrhoids, with no evidence of malignancy. 

The patient’s clinical course improved with antibiotic therapy and supportive care. His respiratory status gradually stabilized, allowing weaning of supplemental oxygen. The hepatic drain was removed on hospital day 8 following decreased output and clinical improvement. 

He was discharged on a prolonged course of oral antibiotics, including levofloxacin 750 mg daily and metronidazole 500 mg every 12 hours, to complete a total of six weeks of therapy from the date of drainage. Oral fluoroquinolone step-down was guided by confirmed in vitro susceptibility of the *Klebsiella pneumoniae* isolate. While a randomized controlled trial demonstrated noninferiority of early oral ciprofloxacin step-down compared to continued intravenous ceftriaxone for KLA, levofloxacin was selected for its once-daily dosing advantage during a prolonged outpatient course and comparable in vitro activity against susceptible isolates. A total duration of six weeks was chosen, given the patient's complicated course, including sepsis, ICU admission, multiloculated abscess with incomplete drainage, and severely uncontrolled diabetes, consistent with the recommended four-to-six-week treatment duration for PLA. A repeat CT scan was planned four weeks after discharge to assess for resolution of the abscess. 

During hospitalization, the patient was initiated on insulin therapy for newly diagnosed type 2 diabetes mellitus, including basal insulin glargine and prandial insulin lispro. He received diabetes education and nutritional counseling, with plans for outpatient endocrinology follow-up for long-term glycemic management. 

## Discussion

This case illustrates a clinically important, but underrecognized, presentation of type 2 diabetes mellitus: KLA may serve as the initial clinical indicator for unmasking previously undiagnosed hyperglycemia. While the patient’s HbA1c of 13.1% confirmed months of severe hyperglycemia, the diagnosis remained hidden until a life-threatening infection necessitated hospitalization. The link between diabetes and KLA is well established. A nationwide cohort study in Taiwan found that patients with newly diagnosed type 2 diabetes carry a nearly threefold increased risk of PLA compared to non-diabetic controls, with the highest risk found in younger males within two years of diagnosis [12]. Furthermore, research demonstrates a dose-response relationship between fasting plasma glucose (FPG) and abscess risk; poorly controlled diabetes (FPG > 130 mg/dL) increases the hazard of infection by 3.34-fold [13]. These data directly mirror the demographics and clinical trajectory observed in this case and reinforce that invasive infections may occasionally represent the first clinical manifestation of previously undiagnosed diabetes mellitus. Clinicians should therefore maintain a high index of suspicion for underlying metabolic disease when evaluating patients with atypical or severe infections. 

The patient’s complications of sepsis and acute hypoxic respiratory failure requiring ICU admission reflect the high morbidity of KLA in diabetic populations. In studies of PLA patients requiring intensive care, diabetes is the most common underlying condition (51%), and acute respiratory failure stands as the strongest independent predictor of mortality [14]. These findings underscore the importance of early recognition and aggressive management in patients with suspected KLA. 

Effective management requires a dual approach of combined source control and antimicrobial therapy. Pigtail catheter drainage, as performed in this case, has been shown to be protective against both metastatic infection and mortality [15]. Early drainage remains a cornerstone of management, even in cases where complete radiologic resolution is not immediately achieved. A randomized controlled trial demonstrated that early transition to oral ciprofloxacin was noninferior to continued intravenous ceftriaxone after a median of five days of effective intravenous therapy, supporting oral step-down when clinical improvement is achieved [16]. 

The pathophysiologic mechanisms described in the introduction directly account for this patient's clinical trajectory. His severely uncontrolled hyperglycemia (HbA1c 13.1%) would be expected to significantly impair neutrophil phagocytosis of K1/K2 serotype Klebsiella pneumoniae, as demonstrated in studies comparing poorly controlled diabetic patients to well-controlled and nondiabetic individuals [9,10]. Moreover, sustained glucose elevations at this level promote capsular polysaccharide biosynthesis in virulent strains, enhancing resistance to opsonophagocytic killing and likely facilitating both hepatic abscess formation and hematogenous dissemination in this case [9,10]. These converging deficits in host defense plausibly explain his progression from occult hepatic infection to bacteremia, sepsis, and respiratory failure despite the absence of any known immunocompromising condition.

Finally, addressing the underlying metabolic disorder is critical to improving outcomes. Poor glycemic control has been associated with increased risk of recurrence and higher short-term mortality in patients with PLA [13,17]. Early initiation of insulin therapy and structured diabetes education during hospitalization, as performed in this case, are essential components of care and may reduce long-term complications. 

Clinical implications

This case reinforces the importance of routine diabetes screening in patients presenting with PLA or other severe invasive infections, even in the absence of a known metabolic history. Current American Diabetes Association (ADA) guidelines recommend screening all asymptomatic adults aged 35 years and older; however, approximately 25% of individuals with type 2 diabetes in the United States remain undiagnosed [18]. HbA1c is particularly valuable in the acute care setting, as it reflects average glycemia over the preceding two to three months and is generally unaffected by acute illness or stress hyperglycemia. However, results should be interpreted cautiously in patients with anemia, hemoglobinopathies, recent transfusion, chronic kidney disease, or altered red blood cell turnover [19]. 

In this patient, an HbA1c of 13.1% confirmed previously undiagnosed, uncontrolled diabetes. In a retrospective study of diabetic patients with KLA, HbA1c level was identified as an independent risk factor for metastatic infection, with cryptogenic abscess and metastatic complications significantly more common in patients with HbA1c >10% [10, 20]. Similarly, poor glycemic control (HbA1c ≥9%) was independently associated with invasive syndrome in diabetic patients with community-acquired K. pneumoniae bacteremia (adjusted OR 5.66; 95% CI 2.01-15.92) [16]. These findings suggest that this patient's degree of hyperglycemia placed him at substantially elevated risk, though HbA1c alone has not been validated as a standalone predictor of metastatic disease or recurrence. Prompt recognition allowed for the early initiation of insulin therapy and diabetes education during the index hospitalization, which may improve long-term adherence and glycemic control. 

Additionally, this case highlights the importance of early and aggressive source control. Percutaneous catheter drainage has been shown to reduce the risk of metastatic infection and mortality in KLA, and timely intervention likely contributed to this patient’s favorable outcome [15]. 

Learning points

Patients presenting with severe or atypical infections, particularly PLA caused by *Klebsiella pneumoniae*, should be screened for diabetes mellitus using HbA1c, as undiagnosed diabetes can contribute to greater disease severity and susceptibility to invasive infection. Early source control via percutaneous drainage should be prioritized when feasible, as it is a cornerstone of effective management. Antimicrobial therapy must be tailored to culture and susceptibility results to ensure appropriate treatment and minimize complications. Additionally, optimizing glycemic control during hospitalization and ensuring outpatient follow-up are essential to reduce the risk of recurrent infection and adverse clinical outcomes.

## Conclusions

*Klebsiella pneumoniae* liver abscess may represent the initial presentation of previously undiagnosed type 2 diabetes mellitus. In this patient, severe chronic hyperglycemia likely contributed to impaired host defenses and the development of invasive infection. Recognition of this association can facilitate earlier diagnosis of diabetes, while prompt source control, culture-directed antimicrobial therapy, and glycemic management remain critical to achieving favorable outcomes. 
